# The Contributions of Pandemic Severity, Government Stringency, Cultural Values and Internet Usage to Post-traumatic Stress Disorder During the COVID-19 Pandemic: An Analysis of Data From 35 Countries

**DOI:** 10.3389/fsoc.2022.881928

**Published:** 2022-05-10

**Authors:** Ravi Philip Rajkumar

**Affiliations:** Department of Psychiatry, Jawaharlal Institute of Postgraduate Medical Education and Research, Puducherry, India

**Keywords:** COVID-19, post-traumatic stress disorder, cultural collectivism, prevalence, mortality rate, case-fatality ratio, government stringency

## Abstract

Emergent symptoms of post-traumatic stress disorder (PTSD) have been frequently reported in the context of the COVID-19 pandemic, and may affect up to 17–18% of individuals. There is preliminary evidence that pandemic severity, cultural values, restrictions imposed by governments, and Internet usage may all influence the emergence of PTSD symptomatology. In this study, possible linear- and non-linear associations between these factors and the prevalence of PTSD symptoms across 35 countries were examined based on data from existing research. Evidence was found for a positive logarithmic relationship between the COVID-19 case-fatality ratio and PTSD (*p* = 0.046), a positive logarithmic relationship between power distance and PTSD (*p* = 0.047), and a trend toward a negative quadratic association with Internet usage (*p* = 0.051). No significant cross-national effect was observed for government restrictiveness. These findings suggest that strategies aimed at minimizing COVID-19 deaths, and at ensuring equitable access to essential resources, may be of use in reducing the emergence of PTSD symptoms at a population level during this pandemic.

## Introduction

The COVID-19 pandemic has been associated with emergent symptoms of psychological distress on an unprecedented scale (Zhang and Chen, 2021). Though some researchers have attempted to group these symptoms under the umbrella of a “COVID stress syndrome” (Taylor, 2021; Taylor et al., 2021), most studies in this field have attempted to measure the frequency and severity of symptoms of “classical” psychiatric syndromes, such as anxiety and depression, using standardized measurement tools (Nochaiwong et al., 2021). A recent global analysis estimated that 33% of individuals developed symptoms of anxiety, 28% symptoms of depression and 30% insomnia related to the pandemic (Liu et al., 2021), suggesting that up to one-third of individuals may have experienced significant psychological distress over the course of the COVID-19 pandemic.

One particular form of psychological distress that has attracted significant attention during the pandemic is post-traumatic stress disorder (PTSD). PTSD is a complex syndrome characterized by persistent symptoms of re-experiencing, avoidance/numbing, and hyperarousal, occurring after exposure to a traumatic event which was associated with feelings of helplessness or fear (Friedman et al., 2011). A “traumatic event” is defined as one that involves actual or threatened death or injury, or a threat to physical integrity, directed at the self or at others (Bryant, 2019). Exposure to such events has been common and widespread during the COVID-19 pandemic (Dutheil et al., 2021). According to the most recently published meta-analysis at the time of writing, the estimated prevalence of significant PTSD symptomatology during the COVID-19 pandemic is 17.5%. However, this estimate included studies of general population samples as well as groups perceived to be at a higher risk of PTSD, such as survivors of severe COVID-19 and healthcare or other frontline workers (Yunitri et al., 2022).

Certain factors have been found to predict the development of symptoms of PTSD during the pandemic. These include demographic factors such as age, geographical location and employment (e.g., nursing staff or other healthcare workers), and methodological factors such as the choice of instrument used to screen for PTSD or the specific group being studied (e.g., patients with COVID-19, staff working in COVID-19 units); however, the differences that could be attributed to these factors were modest (Cénat et al., 2021; Yunitri et al., 2022). However, certain other factors that were found to contribute to psychological distress during the pandemic have not been specifically studied in the context of PTSD. These include the local severity of the pandemic (COVID-19 Mental Disorders Collaborators., 2021), the severity of restrictions on human mobility (COVID-19 Mental Disorders Collaborators., 2021; Jin et al., 2021), and the prevailing cultural values in a given country, particularly the cultural dimension of individualism vs. collectivism (Shekriladze et al., 2021; Xiao, 2021).

Though these factors have not been fully studied in relation to PTSD during the pandemic, there is translational and empirical evidence of their relevance. For example, quarantine or isolation has been found to contribute to PTSD during earlier outbreaks of infectious disease (Reynolds et al., 2008; Henssler et al., 2021); forced quarantine may be more traumatic than voluntary quarantine (TMGH-Global COVID-19 Collaborative., 2021); emergent PTSD has been noted at higher rates in regions with a higher COVID-19 incidence rate (Carmassi et al., 2022); and sudden death of a loved one due to COVID-19, or enforced separation from them prior to death due to infection control measures, can contribute to traumatic grief (Masiero et al., 2020; Djelantik et al., 2021). It has also been observed that cultural values such as collectivism shape the appraisal of trauma and influence both the emergence and persistence of PTSD symptoms (Jobson, 2009). Culture can also influence the level of social support provided to those exposed to pandemic-related traumatic stress (Messner, 2021), and this can protect against the emergence of post-traumatic stress (Gentry et al., 2022). Finally, several dimensions of culture are associated with the transmission of SARS-CoV-2, and this might exert an indirect effect on PTSD through the traumatic effects of quarantine, hospitalization, or bereavement (Chen and Biswas, 2022; Duarte et al., 2022).

The COVID-19 pandemic has also been characterized by high rates of online and social media usage, amplified by restrictions on in-person social contact. There is some evidence of a link between higher social media usage and PTSD symptomatology. This may be due to the “sensitizing” effect of repeated exposure to pandemic-related images and stories on vulnerable individuals exposed to pandemic-related traumatic stressors (Ikizer et al., 2021; Price et al., 2022).

In the light of the above findings, the current study attempted to examine the relative contributions of COVID-19 severity indicators, the stringency of governmental responses to the pandemic, the national level of cultural collectivism, and national Internet usage on cross-national variations in the prevalence of significant symptoms of PTSD.

## Methodology

In this study, cross-national variations in the prevalence of PTSD symptoms were examined in relation to three indices of COVID-19 severity (prevalence, crude mortality rate and case-fatality ratio), a culturally neutral index of individualism-collectivism (the Global Collectivism Index), a standardized measure of the restrictiveness of governmental measures to control the pandemic (the COVID-19 Government Stringency Index), and a proxy measure of national internet usage (the percentage of individuals using the Internet in each country), while correcting for methodological factors that could independently affect the prevalence of PTSD symptoms, such as age and gender distributions of study samples or the nature of the screening tool used. For the purpose of this study, “prevalence of PTSD symptoms” was defined as the percentage of individuals scoring above a specified cut-off for clinical concern on a standardized screening instrument for PTSD. This figure denotes the proportion of individuals who screened positive in a given study, and should not be understood as a measure of the prevalence of syndromal PTSD.

### Data Sources

#### PTSD Prevalence and Methodological Factors

A literature search of the PubMed, Scopus and ProQuest databases was carried out using the search terms (“COVID-19”, “COVID”, “SARS-CoV-2”, either alone or joined to “pandemic”) AND (“PTSD”, “post-traumatic stress disorder”, “post-traumatic stress”, “post-traumatic stress symptoms”). After screening a total of 1,051 citations, 20 relevant studies covering 35 countries were included in the analysis. Studies were included only if they (a) involved subjects from general population samples, (b) provided a quantitative estimate of the frequency of PTSD at a specific point in time, and (c) used a standardized and validated screening tool or instrument for the identification of clinically significant PTSD symptomatology. General population studies were selected for analysis to minimize the number of potential confounders that might arise if “high-risk” populations, such as frontline healthcare workers and COVID-19 survivors, were sampled. All studies included in this paper were based on data collected during the year 2020. The estimated prevalence of PTSD symptoms (PTSD-Prev), expressed as a percentage, was the dependent variable in the current study.

For each study, the following methodological variables were also extracted: (a) nature of the screening instrument used, (b) sample size, (c) time between the onset of the pandemic in the concerned country and the collection of data, measured in months, (d) mean age of the study sample, and (e) gender distribution, expressed as percentage of female participants in the sample. These factors were selected based on observations that they might influence estimates of the frequency of PTSD symptoms in earlier reviews and meta-analyses.

When designing this study, a comparison of studies measuring the prevalence of PTSD symptoms in 2020, 2021 and 2022 was envisaged. However, a comprehensive review of literature revealed that though there were studies published in 2021 and 2022, most of these either: (a) reported data from 2020, (b) did not provide a percentage of the number of individuals who screened positive, or (c) were focused on specific high-risk populations, such as healthcare workers, individuals hospitalized for severe COVID-19, or people with a pre-existing mental illness. As these studies could not be compared to those conducted in general population samples, this part of the study could not be carried out.

#### COVID-19 Severity Indices

Three indices of the severity of the COVID-19 pandemic were examined for each country. The estimated prevalence (C19-Prev) is defined as the number of confirmed COVID-19 cases per 1 million population, while the crude mortality rate (CMR) is defined as the number of confirmed deaths due to COVID-19 per 1 million population, and the case-fatality ratio (CFR) is the ratio of deaths to total cases of COVID-19, expressed as a percentage. Though these measures have certain inherent limitations due to variations in testing, reporting and death certification practices, they have been widely used to quantify the severity of the pandemic at a cross-national level (Favas et al., 2022). Information on these variables was obtained from the Johns Hopkins University's global COVID-19 data aggregator. For each study, data on COVID-19 severity was collected and entered for the time at which the individual study was conducted (Johns Hopkins Coronavirus Resource Center., 2022).

#### Global Collectivism Index (GCI)

Though several measures of cultural individualism-collectivism have been described in the literature, their validity is open to question as they are mostly based on data obtained from Western, industrialized countries with a democratic form of government. To address this, the GCI was developed to provide a truly global estimate of cultural collectivism, based on data from 188 nations, including several Asian and African countries that were excluded in earlier analyses (Pelham et al., 2022). A positive GCI indicates a collectivist culture (the highest being Somalia, with a GCI of 1.92), while a negative GCI indicates a more individualist culture (the lowest being Monaco, with a GCI of −1.85). The CGI shows moderate to high positive correlations with all prior measures of cultural collectivism.

#### Other Cultural Dimensions (Hofstede)

A review of the existing literature found that, besides individualism-collectivism, three cultural dimensions appeared to correlate with COVID-19 transmission. These dimensions were power distance, masculinity-femininity and uncertainty avoidance. Power distance reflects the extent to which a society follows a strict hierarchy and accepts inequalities; this parameter was associated with adherence to government restrictions (Messner, 2021) and reduced numbers of hospital or ICU admissions (Duarte et al., 2022). Masculinity-femininity measures the extent to which a society is oriented toward achievement, assertiveness and competition, as opposed to cooperation and nurturing; high masculinity scores are associated with the number of COVID-19 cases and deaths at a national level (Chen and Biswas, 2022). Uncertainty avoidance indicates the extent to which a society is able to tolerate ambiguous or uncertain situations, with high scores indicating lower tolerance; high uncertainty avoidance is also associated with increased COVID-19 prevalence and mortality (Chen and Biswas, 2022). Therefore, these three cultural dimensions were also included in the analysis. Data on these variables was obtained from the Hofstede Insights database (Hofstede Insights, 2022).

#### Government Stringency Index (GSI)

Governments across the world have varied in the extent, severity and duration of the restrictions imposed on their subjects during the COVID-19 pandemic. The GSI, computed by the Oxford Coronavirus Government Response Tracker (OxCGRT) provides a composite measure of all these restrictions, including school and work closures, restrictions on public events and gatherings, quarantine measures and restrictions on internal and external travel. The GSI can take on any value from 0 to 100, with 0 indicating the least stringent response and 100 indicating the most stringent response (Hale et al., 2021; Our World In Data., 2022). For the purpose of this study, the estimated GSI for the time at which each individual study was conducted was included in this analysis. For example, if a study of pandemic-related PTSD was conducted in May 2020, the GSI as of May 31, 2020 was entered in the corresponding row for that study.

#### Internet Usage

As there is no reliable, large-scale estimate of social media usage at a cross-national level, the percentage of Internet users per country was utilized as a proxy measure for time spent consuming online or social media. Information on this variable was obtained from the World Bank's database and is based on aggregated data from telecommunication unions (World Bank, 2022).

#### Data Analysis

Data analysis was carried out using the Statistical Package for Social Sciences, version 20.0 (SPSS version 20.0, SPSS Inc.) All study variables were tested for normality prior to data analysis. Three key study variables—CMR, CFR and GCI—did not conform to a normal distribution (*p* < 0.01, Shapiro-Wilk test).

In the first step of the analysis, the association between the five methodological variables listed above and the estimated prevalence of PTSD symptoms in each study was examined as follows: For continuous variables such as sample size and mean sample age and time, bivariate correlations (Pearson's and Spearman's) were computed to assess the possibility of a linear or monotonic association between these variables and PTSD symptoms. To assess the effect of the screening tool used, a one-way analysis of variance (ANOVA) was carried out, followed by a *post-hoc* Bonferroni test, to examine whether PTSD symptom prevalence differed significantly across studies using different tools.

In the second step, bivariate correlations between PTSD-Prev and the independent variables of interest were examined using Pearson's and Spearman's correlation analyses. Both methods were used in parallel in view of the possible non-normal distribution of the aforementioned variables. Finally, attempts were made to test for non-linear relationships between PTSD-Prev and the independent variables using the curve estimation function for logarithmic and quadratic models if the visual inspection of scatter plots suggested such a relationship, and/or if the monotonic model suggested a trend toward an association. These plots are provided in the Supplementary Material. All statistical tests were two-tailed, and a significance level of *p* < 0.05 was considered significant. In view of the exploratory nature of this study and its small sample size, correction for multiple comparisons was not undertaken.

## Results

Data on PTSD-Prev could be retrieved for a total of 35 countries, based on 23 published studies. A complete description of these studies and their methodological characteristics is provided in the Supplementary Material. The estimated prevalence of PTSD symptoms ranged from a minimum of 11.7% in a Vietnamese sample to 49.6% in an Iranian sample, with a mean prevalence of 29.8 ± 10.2%.

### Analyses of Methodological Factors

There were significant variations in sample size, time of sampling, age, and gender distribution across the included studies. Moreover, a variety of instruments were used to estimate PTSD symptom severity. The most commonly used instrument was the Primary Care PTSD Screen for DSM-5 (PC-PTSD-5, 13 countries) followed by the PTSD Checklist for DSM-5 (PCL-5, 12 countries), the Impact of Event Scale-Revised (IES-R, 5 countries), the International Trauma Questionnaire (ITQ, 3 countries), the Startle, Physiological Arousal, Anger and Numbness screening instrument (SPAN, one country) and the Screen for Post-Traumatic Stress Symptoms Tool for DSM-IV (SPTSS, one country). No significant correlation could be identified between the prevalence of PTSD symptoms and either sample size (*r* = −0.17, *p* = 0.324), time gap between the imposition of pandemic measures and sample evaluation (*r* = −0.05, *p* = 0.947), mean age of the study sample (*r* = −0.20, *p* = 0.242) or gender distribution (*r* = −0.19, *p* = 0.288). No multicollinearity could be identified between any of the methodological variables themselves (*r* < 0.5 for all correlations). There was a significant effect of the choice of study instrument on PTSD-Prev (*F* = 3.4, *p* = 0.021). On *post-hoc* analysis, the only significant inter-instrument difference noted was between studies using the ITQ and studies using the SPAN or SPTSS (*p* = 0.028, Bonferroni *post-hoc* test), with a similar trend between the ITQ and PCL-5 (*p* = 0.087, Bonferroni *post-hoc* test). No significant difference could be identified between any of the other instruments. From this analysis, it was evident that only the ITQ appeared to significantly influence variations in PTSD symptom estimates. To account for this, subsequent analyses were conducted both with the entire sample (*n* = 35) and after excluding studies which had used the ITQ (*n* = 32).

### Analyses of Pandemic Severity Indices

Correlations between the three indices of COVID-19 severity (prevalence, crude mortality rate, and case fatality rate) are presented in Table 1. It can be seen from these results that none of these variables showed a significant linear or monotonic correlation with PTSD-Prev. However, there was a significant and positive correlation between the prevalence of PTSD symptoms and the logarithm of the COVID-19 case fatality rate (*r* = 0.34, *R*^2^ = 0.12, *p* = 0.046). No significant association could be identified for any of the other COVID-19 severity indices. There was no significant multicollinearity between any of the COVID-19 indices themselves (*r* < 0.6 for all correlations). When these analyses were repeated with the subset of studies not using the ITQ, these results were not altered substantially.

**Table 1:** Linear and non-linear relationships between COVID-19 severity indices and the estimated prevalence of PTSD symptoms by country.

**Variable**	**Linear correlation (*r)***	**Monotonic correlation (ρ)**	**Evidence for non-linear correlation in scatter plot or trend**	**Non-linear association, if applicable**
**C19-Prev**	−0.23 (0.186)	−0.19 (0.279)	None	N/A
**CMR**	−0.01 (0.979)	0.16 (0.375)	None	N/A
**CFR**	0.23 (0.191)	0.28 (0.105)	Yes	Logarithmic *r* = 0.34^*^ p = 0.046

*C19-Prev, estimated national prevalence of COVID-19; CMR, estimated COVID-19 crude mortality rate; CFR, COVID-19 case-fatality ratio; r, Pearson's correlation coefficient; ρ, Spearman's correlation coefficient; N/A, not applicable*.

* *Significant at p < 0.05*.

### Analysis of Cultural Variables, Stringency, and Internet Usage

Correlations between PTSD-Prev, CGI and CGI scores, and percentage of Internet users are presented in Table 2. Linear analyses revealed trend-level associations of a positive nature for power distance (*r* = 0.30, *p* = 0.084) and of a negative nature for Internet usage (*r* = −0.33, *p* = 0.052). Non-linear models found a significant positive association between the natural logarithm of power distance and PTSD-Prev (*r* = 0.34, *p* = 0.047); however, the association with Internet usage remained at a trend level. No significant linear or non-linear correlation between other cultural dimensions or government stringency and PTSD-Prev could be identified.

**Table 2:** Linear and non-linear associations between cultural dimensions, government stringency, internet usage and the estimated prevalence of PTSD symptoms by country.

**Variable**	**Linear correlation (*r)***	**Monotonic correlation (ρ)**	**Evidence for non-linear correlation in scatter plot or trend**	**Non-linear association (best fit), if applicable**
Individualism-collectivism	0.22 (0.208)	0.22 (0.209)	None	N/A
Power distance	0.30 (0.084)	0.33 (0.051)	Yes	Logarithmic *r* = 0.34 *p* = 0.047
Masculinity-femininity	0.22 (0.208)	0.15 (0.392)	None	N/A
Uncertainty avoidance	0.25 (0.149)	0.25 (0.15)	None	N/A
Government stringency	0.04 (0.814)	0.09 (0.598)	None	N/A
Internet usage	−0.33 (0.052)	−0.33 (0.056)	Yes	Quadratic *R^2^* = 0.11 *p* = 0.051

*Abbreviations: N/A, not applicable*.

### Intercorrelations Between Cultural and Other Variables

As an additional measure, correlations between cultural dimensions and the other independent variables of interest (COVID-19 indices, government stringency, and Internet usage) were examined. Cultural collectivism was positively correlated with both stringency (*r* = 0.61, *p* < 0.01) and C19-CFR (*r* = 0.*5*1, *p* < 0.01) and negatively correlated with C19-Prev (*r* = −0.68, *p* < 0.01) and Internet usage (*r* = −0.68, *p* < 0.01). An identical pattern of correlations was obtained for power distance (stringency: *r* = 0.41, *p* = 0.014; C19-CFR: *r* = 0.45, *p* < 0.01; C19-Prev, *r* = −0.61, *p* < 0.01; Internet usage, *r* = −0.58, *p* < 0.01). Masculinity was positively correlated with stringency (*r* = 0.36, *p* = 0.034), while uncertainity avoidance was positively correlated with C19-CMR (*r* = 0.45, *p* < 0.01).

### Partial Correlation Analyses

As C19-CFR appeared to be independently associated with PTSD prevalence and with the cultural dimensions of collectivism and power distance, partial correlation analyses between these two dimensions and PTSD prevalence were conducted with C19-CFR held constant. However, neither of these associations were statistically significant (power distance x PTSD: *r* = 0.*2*2, *p* = 0.203; collectivism x PTSD: *r* = 0.*1*2, *p* = 0.489).

In view of the lack of significant bivariate linear analyses, multivariate linear regression was not attempted.

## Discussion

Post-traumatic stress disorder during the COVID-19 pandemic has been the focus of intense debate and research. While some researchers have warned of a “second pandemic” of PTSD in the wake of the damage caused by this pandemic (Dutheil et al., 2021), others have highlighted the heterogeneity of psychological responses to COVID-19, as evidenced by both cross-national variations in the prevalence of psychological distress (Shevlin et al., 2021) and the phenomena of post-traumatic growth and resilience which mitigate against the persistence of PTSD symptoms (Killgore et al., 2020; Gonda and Tarazi, 2022). The current study suggests that the “heterogeneity” view may be closer to reality than the “tsunami” view, as a wide range of reported rates of PTSD symptoms was observed across the studies analyzed in this paper. However, even the lowest reported rate included in this study (11.5%) is comparable to the estimate of 17-18% reported in meta-analyses, suggesting that a significant minority of the general population experiences PTSD symptoms in response to the pandemic. There is insufficient evidence to comment on what proportion of these individuals will continue to experience chronic PTSD; past evidence (Mak et al., 2009; Wang et al., 2020) suggests that these symptoms may diminish or resolve over time in some cases, while current studies have yielded equivocal results (Benfante et al., 2022; Kalaitzaki et al., 2022).

In this study, we identified a possible association between the prevalence of PTSD symptoms and the COVID-19 case-fatality ratio. This finding is significant in the light of the debate surrounding the nature of traumatic stressors during the COVID-19 pandemic. While some authors have argued for a rigorous definition which would include only severe events (involving personal or occupational exposure to death or the risk of death) as “traumatic stress”, others have suggested considering “pandemic exposure”, or events such as being placed in quarantine or subjected to movement restrictions, as traumatic events *per se* (Norrholm et al., 2021). This debate is an extension of ongoing discussions of whether the spectrum of “traumatic events” is being unduly broadened (Jones, 2021). The current results, though subject to certain important limitations, suggest that the “narrower” definition of traumatic stress may be accurate, as the case-fatality ratio is a reflection of the risk of death in an infected individual, as opposed to measures such as prevalence (which includes mild and asymptomatic cases).

Among cultural dimensions, power distance showed a tentative positive association with the prevalence of PTSD symptoms. Societies with high power distance are characterized by institutionalized inequality. In the context of the COVID-19 pandemic, unequal distribution of healthcare and other resources may have contributed to traumatic stress in the general population. Power distance was associated with an increased COVID-19 case fatality ratio in this study, which could have contributed to traumatic grief. It is possible that other sociocultural factors, which were not analyzed in this study may also influence the emergence of post-traumatic stress symptoms (Ohta et al., 2021). It is also possible that these factors exert a greater influence on other forms of psychological distress, such as depression and anxiety, than on PTSD.

Though a negative association between Internet usage and PTSD symptoms was observed in this analysis, this finding was just below the threshold for statistical significance. While certain aspects of Internet usage, such as consumption of pandemic-related media (“doomscrolling”) have been associated with PTSD (Price et al., 2022), the Internet may also be used for social connection, communication of vital information, purchase of essentials and even healthcare (“telemedicine”) during periods of confinement or isolation (Farsi et al., 2022). The role of the Internet in shaping positive or negative psychological responses to COVID-19 is complex, and requires further elucidation along multiple vectors in diverse populations.

As discussed earlier, the planned comparison of studies from 2020, 2021 and 2022 could not be carried out due to the low number of studies sampling subjects in 2021 and 2022. In a study of four countries (Germany, Israel, Poland and Slovenia), both government stringency and the percentage of subjects “at risk” of PTSD fell by around 5% between February and June 2021; however, the authors did not test for a significant association between these variables (Benatov et al., 2022). In contrast, a study of the Italian general population found a non-significant increase in PTSD symptomatology from April 2020 (19%) to January 2021 (21%); no specific demographic variables were associated with changes in PTSD symptoms at the individual level (Benfante et al., 2022). In contrast, a study from Greece comparing the frequency of PTSD symptoms during two successive lockdowns (March–May 2020 and November 2020–May 2021) found a significant increase in symptoms (36% vs. 26%) during the second lockdown (Kalaitzaki et al., 2022). The variability of these results highlights the need for further multi-country longitudinal research in this field, with an analysis of both individual and broader social and cultural factors.

This study is subject to certain important limitations. It is based on data derived from various studies, and this may lead to variations due to methodological factors, despite the efforts made to address these in the current study. It is largely derived from data obtained during the first “wave” of the COVID-19 pandemic, and these findings may not generalize to PTSD emerging at a later stage of this pandemic. It is cross-sectional and correlational in nature, meaning that no firm conclusions regarding causality can be drawn. It is based on data from a limited number of countries, meaning that information from certain geographical areas, such as sub-Saharan Africa and Oceania, was not available. Though certain significant findings were obtained, these require replication and may not survive more rigorous forms of statistical correction. As they were based on national-level data, these findings are not directly applicable to individuals. Other factors that may significantly influence the emergence of pandemic-related PTSD, such as prior physical and mental health status, or increases in intimate partner violence, could not be assessed (Thibaut and van Wijngaarden-Cremers, 2020). The dependence of the study findings on published data imply that they are sensitive to publication bias. No correction was made for multiple comparisons in the correlation analyses, raising the possibility of false-positive findings. Finally, as this study was based on a secondary analysis of earlier research, it could not be registered in a database of prospective observational, interventional or meta-analytic studies.

## Conclusion

Despite the above limitations, this study suggests that significant relationship may exist between the COVID-19 case fatality rate and the emergence of PTSD symptoms in the context of the pandemic. Provisional evidence of a positive association with cultural power distance and a negative association with Internet usage were also observed. These findings suggest that interventions aimed at improving COVID-19 survival (such as high-risk prevention strategies and prompt treatment of severely ill patients) may foster resilience and reduce the emergence of PTSD at the level of the general population. It is also possible that attempts to ensure equitable access to essential resources may also reduce this risk in societies with high levels of inequality (Condon et al., 2020), but this recommendation should be considered tentative.

## Data Availability Statement

The original contributions presented in the study are included in the article/Supplementary Material, further inquiries can be directed to the corresponding author/s.

## Author Contributions

The author confirms being the sole contributor of this work and has approved it for publication.

## Conflict of Interest

The author declares that the research was conducted in the absence of any commercial or financial relationships that could be construed as a potential conflict of interest.

## Publisher's Note

All claims expressed in this article are solely those of the authors and do not necessarily represent those of their affiliated organizations, or those of the publisher, the editors and the reviewers. Any product that may be evaluated in this article, or claim that may be made by its manufacturer, is not guaranteed or endorsed by the publisher.
