# Supplementary figures and images for: The Contributions of Pandemic Severity, Government Stringency, Cultural Values and Internet Usage to Post-traumatic Stress Disorder During the COVID-19 Pandemic: An Analysis of Data From 35 Countries
Source: Front Sociol. 2022 May 10;7:881928. doi: 10.3389/fsoc.2022.881928 (PMC9127200; doi:10.3389/fsoc.2022.881928)

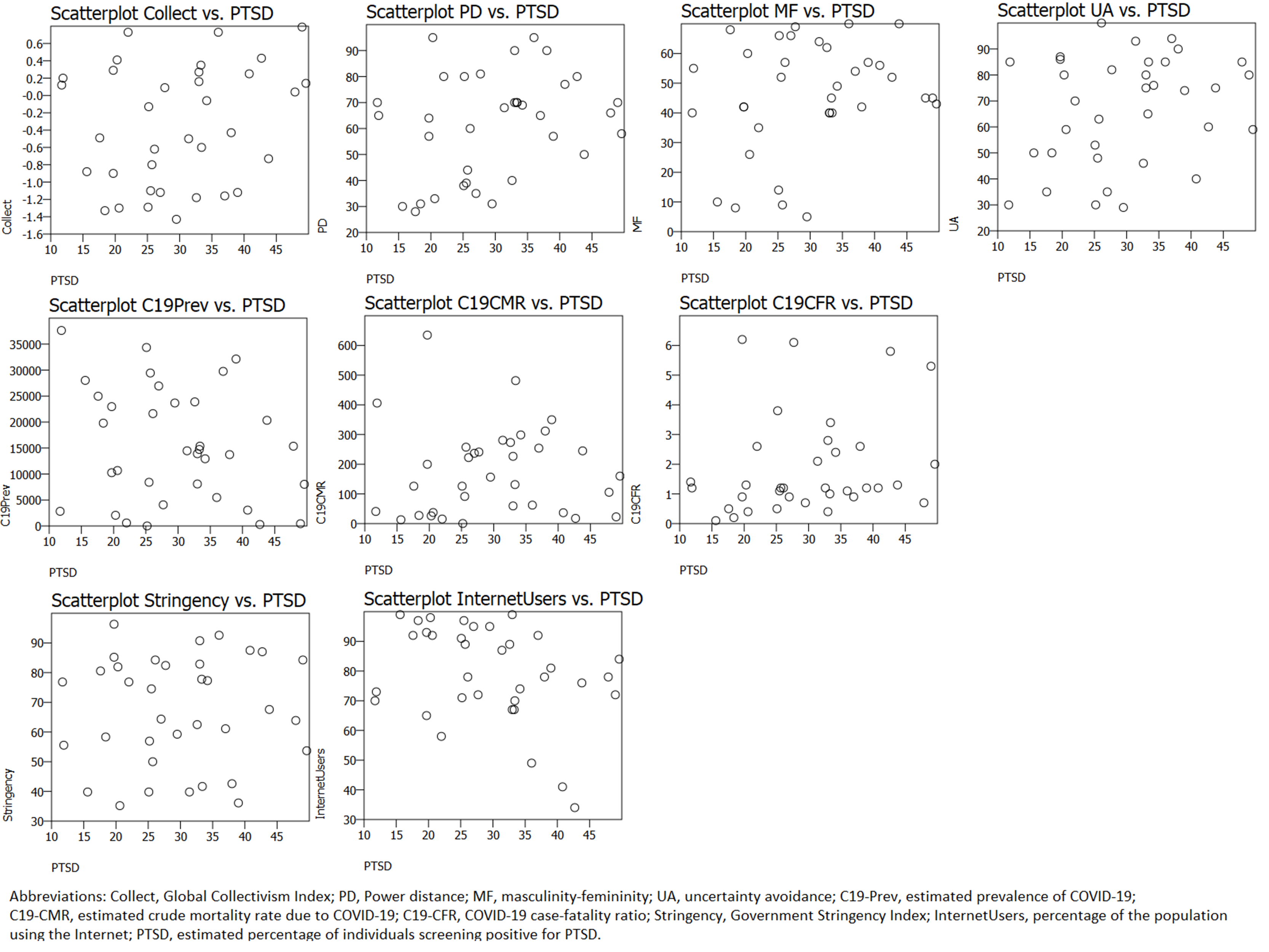

Supplement: Supplementary file 1 [file Image_1.PNG]
